# Diversity and Evolution of *NLR* Genes in *Citrus* Species

**DOI:** 10.3390/biology13100822

**Published:** 2024-10-14

**Authors:** Zhiwei Xiong, Wanshan Zhang, Hui Yin, Jiaxing Wan, Zhuozhuo Wu, Yuxia Gao

**Affiliations:** 1 National Navel Orange Engineering Research Center, Gannan Normal University, Ganzhou 341000, China; xzw@gnnu.edu.cn (Z.X.); zws@gnnu.edu.cn (W.Z.); yh@gnnu.edu.cn (H.Y.); wjx@gnnu.edu.cn (J.W.); three_kangaroo@163.com (Z.W.); 2 Jiangxi Provincial Key Laboratory of Pest and Disease Control of Featured Horticultural Plants (2024SSY04181), Ganzhou 341000, China

**Keywords:** *NLR* genes, *citrus*, diversity, adaptive evolution

## Abstract

**Simple Summary:**

*Citrus* (*Citrus* spp.) is the world’s largest fruit crop, both in terms of cultivation area and production. In recent years, the *citrus* industry in countries such as China, Brazil, and the United States has suffered significant damage due to the increasing severity of *citrus* diseases, such as *Citrus* Huanglongbing (HLB). Plant nucleotide-binding leucine-rich repeat (*NLR*) genes play a critical role in the plant immune system by helping plants resist pathogen infections. A thorough understanding of the diversity and evolution of *citrus NLR* genes is of great significance for the development of disease-resistant cultivars. In this study, we systematically identified 1585 *NLR* genes across 10 *citrus* genomes and conducted an in-depth analysis of their origins and evolutionary processes.

**Abstract:**

*NLR* genes are crucial components of the effector-triggered immunity (ETI) system, responsible for recognizing pathogens and initiating immune responses. Although *NLR* genes in many plant species have been extensively studied, the diversity of *NLR* genes in *citrus* remains largely unknown. Our analysis revealed significant variations in the copy numbers of *NLR* genes among these species. Gene duplication and recombination were identified as the major driving forces behind this diversity. Additionally, horizontal gene transfer (HGT) emerged as the principal mechanism responsible for the increase in *NLR* gene copy number in *A. buxifolia*. The *citrus NLR* genes were classified into four categories: TIR-NBS-LRR (TNL), CC-NBS-LRR (CNL), RPW8-NBS-LRR (RNL), and NL. Our findings indicate that TNL, RNL, and CNL genes originated from NL genes through the acquisition of TIR and RPW8 domains, along with CC motifs, followed by the random loss of corresponding domains. Phylogenetic analysis suggested that *citrus NLR* genes originated alongside the species and underwent adaptive evolution, potentially playing crucial roles in the global colonization of *citrus*. This study provides important insights into the diversity of *citrus NLR* genes and serves as a foundational dataset for future research aimed at breeding disease-resistant *citrus* varieties.

## 1. Introduction

When pathogens infect plants, the plants deploy two innate immune systems—pathogen-associated molecular pattern-triggered immunity (PTI) and effector-triggered immunity (ETI)—to defend against the invasion of pathogens [1,2]. PTI triggers fundamental immune mechanisms such as ethylene production, oxidative bursts, callose deposition, and the expression of defense-related proteins [3,4], thereby impeding the invasion and spread of pathogens. However, pathogens evolve various strategies to counteract or evade PTI defenses. When pathogens release effectors to suppress PTI, the plant’s resistance genes (*R* genes) can directly or indirectly recognize these effectors, activating ETI immune responses. ETI typically leads to a hypersensitive response (HR) at the infection site, where cells rapidly undergo programmed cell death, limiting the further spread of pathogens [5,6]. These two immune systems are interconnected, working together to maintain the health and survival of plants [2,7,8].

*R* genes are critical components of the ETI immune system, playing a key role [6]. R-proteins act as receptors for specific pathogens. Upon recognizing pathogen effectors, these proteins trigger complex signaling pathways that lead to various defense responses, such as the production of antimicrobial compounds, reinforcement of cell walls, and activation of programmed cell death. This rapid response not only limits pathogen spread but also induces systemic acquired resistance (SAR), providing long-lasting immunity against future infections [9]. The *Rps2* gene in *Arabidopsis thaliana* confers resistance to the bacterial pathogen *Pseudomonas syringae*. The Rps2 protein detects the effector AvrRpt2, initiating a hypersensitive response characterized by localized cell death that restricts pathogen growth [10]. Similarly, the *Pi-ta* gene in rice (*Oryza sativa*) provides resistance to the rice blast fungus *Magnaporthe oryzae*. The Pi-ta protein recognizes the effector Avr-Pita, triggering defense responses and effectively curbing the disease [11].

Most *R* genes encode disease resistance proteins with nucleotide-binding site and leucine-rich repeat (NBS-LRR) domains; these genes are thus often termed *NLR* genes [12]. Beyond the canonical NBS and LRR domains, the N-termini of R proteins frequently feature additional domains or motifs, such as the toll/interleukin-1 receptor (TIR) domains, the powdery mildew resistance protein 8 (RPW8) domains, and coiled-coil (CC) motifs [1,13]. Proteins harboring the TIR and RPW8 domains are classified as TNL and RNL, respectively [13,14]. Similarly, proteins with CC motifs are designated as CNL [12]. A significant proportion of R proteins, possessing only the NBS and LRR domains, are collectively categorized as NL (NBS-LRR).

Comparative genomics research has shown that *NLR* genes are extensively distributed among land plants and charophytes, indicating a deep evolutionary origin [1,13,15]. Additionally, *NLR* genes have been identified in a limited number of green algae species, further supporting their ancient lineage [16]. Phylogenetic analyses suggest that the emergence of *R* genes preceded the evolutionary split between charophytes and land plants [1,13]. Following their origin, *NLR* genes have undergone adaptive evolution, significantly contributing to the transition of plants from aquatic to land habitats [13].

*Citrus* is one of the world’s most significant fruits, providing essential nutrients such as vitamin C and citric acid. The cultivation of *citrus* spans over 140 countries and regions globally, thriving in warm and moist climates below the northern latitudes [17]. In 2020, the global *citrus* cultivation area reached 521,000 hectares, yielding a production of 96.81 million metric tons [18]. However, *citrus* faces various threats from diseases, such as *citrus* HLB, a devastating ailment that severely impacts the yield and quality of *citrus*. Addressing this challenge necessitates a deep understanding of *citrus*’s mechanisms of disease resistance. The study of the diversity of disease-resistant genes is foundational for elucidating these defense mechanisms. Currently, there is no systematic research reported on *citrus NLR* genes.

This study integrates comparative genomics and phylogenetics to systematically explore the *NLR* genes in the *citrus* genome, unraveling the mechanisms underlying the diversity, origin, and evolutionary patterns of *citrus NLR* genes. We observed substantial diversity in *NLR* genes among different *citrus* species. Further in-depth investigation revealed that gene duplication and recombination are the primary mechanisms shaping the diversity of *NLR* genes in *citrus*. This study holds significant implications for a profound understanding of *citrus NLR* genes and for breeding disease-resistant *citrus* varieties.

## 2. Materials and Methods

### 2.1. Data Used in This Study

At the outset of this study (September 2020), genomic data for 10 *citrus* species were accessible, namely *C. medica*, *C. ichangensis*, *C. hindsii*, *Atlantia buxifolia*, *C. trifoliata*, *C. grandis*, *C. mangshanensis*, *C. sinensis* Osbeck cv. Newhall, *C. sinensis* Osbeck cv. Valencia, and *C. clementina*. The genome of *C. sinensis* Osbeck cv. Newhall was assembled and annotated by our research team [19] and available for download from the Zenodo database (https://zenodo.org/record/8176803, accessed on 1 January 2020). Genomic data for the remaining 9 *citrus* species can be obtained from the CPBD (http://citrus.hzau.edu.cn/download.php, accessed on 6 September 2024) [20].

### 2.2. Identification of NLR Genes

The nine seed sequences of the NB-ARC domain (PF00931) were obtained from the European Bioinformatics Institute (EBI) database and stored in Stockholm format. A hidden Markov model (HMM) of NB-ARC was constructed using HMMER package 3.1b2 [21]. Then, this model was used to search for proteins containing the NB-ARC domain among the protein sequences of 10 *citrus* genomes (Table S1). The Blastp algorithm was also employed to search for *citrus* proteins containing the NB-ARC domain, using the NLR proteins of *Arabidopsis thaliana* [13] as queries, with a cutoff E-value of 10^−3^. The results obtained from both methods were merged and redundant sequences were removed to obtain *citrus* proteins containing the NB-ARC domain. Multiple sequence alignment of *citrus* proteins containing the NB-ARC domain and previously published NB-ARC or NAT domain proteins from both eukaryotes and prokaryotes [22] was performed using MAFFT v7.526 software [23] with the G-INS-i algorithm, followed by manual editing using MEGA11 [24]. ModelFinder [25] was utilized to search for the best model, and IQ-TREE v2.3.6 software [26] was employed with the JTT+R10 model for phylogenetic analysis. Proteins with MalT and SWACOS domains were used as outgroups [22]. The protein domain architecture was annotated using CD-Search [27]. *NLR* genes were searched in the nucleotide sequences of 10 *citrus* genomes using NLR-Annotator software v2.1 [28] with default parameters for testing analysis of the identification results.

### 2.3. Phylogenetic Analysis

To investigate the origin of *NLR* genes in *citrus*, 1585 identified NLR proteins of *citrus* and 1583 published NLR proteins from the Anacardiaceae and Sapindaceae families were aligned using MAFFT v7.526 [23] with the G-INS-i algorithm, manually edited using MEGA11 [24]. ModelFinder [25] was used to research the best model, and then Q.mammal+R10 was employed for phylogenetic tree construction using IQ-TREE v2.3.6 [26].

The *citrus* NLR gene sequences were aligned using MAFFT v7.526 [23] for multiple sequence alignment, followed by manual editing using MEGA11 [24]. ModelFinder [25] was employed to identify the best model, and subsequently, JTT+F+R10 was used for phylogenetic tree construction using IQ-TREE v2.3.6 [26]. The phylogenetic analysis of *citrus* species based on whole genomes was conducted using the STAG algorithm implemented in OrthoFinder2 [29].

### 2.4. Positive Selection Analysis

Due to the significant divergence of *NLR* genes, alignment and positive selection analyses were not feasible for all sequences. Consequently, we divided them into 65 groups based on phylogenetic analysis. Nucleotide sequences were aligned using MAFFT v7.526 [23] and manually refined with MEGA11 [24]. A likelihood ratio test for positive selection was conducted by comparing the M7 (beta) and M8 (beta and v) models using the PAML v4.9 software package [30,31]. For gene groups demonstrating positive selection via the likelihood ratio test, positively selected sites were identified through the Bayes empirical Bayes (BEB) procedure.

### 2.5. Recombination Analysis

Recombination was detected using the RDP 5.05 package [32]. The aligned nucleotide sequences were imported into RDP 5.05 and analyzed with the RDP, GENECONV, Chimaera, MaxChi, BootScan, SiScan, and 3Seq methods. Potential recombination events were deemed significant only if supported by at least four methods with a *p*-value < 10^−6^ [33].

## 3. Results

### 3.1. Identification and Diversity of Citrus NLR Genes

**Figure 1:**
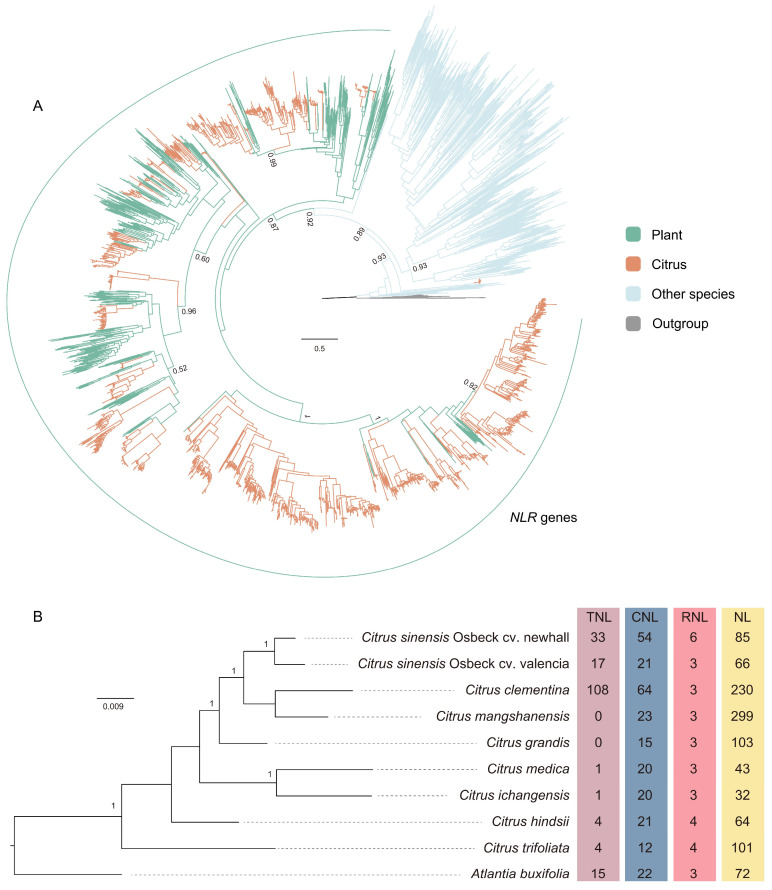
The identification and distribution of *NLR* genes in *citrus*. (**A**) Phylogenetic analysis of NB-ARC domain-containing proteins. Phylogenetic analysis was conducted using an approximate maximum likelihood method with the JTT+R10 model. Proteins with MalT and SWACOS domains were used as outgroups. UFBoot values are shown near the selected nodes. (**B**) The distribution of NLR proteins in 10 *citrus* species. The distribution and number of NLR proteins, including TNL, CNL, RNL, and NL, in various *citrus* species are displayed in the right columns. The phylogenetic relationships among *citrus* species were constructed based on all *citrus* genes.

This study employed comparative genomics and phylogenetics to identify *NLR* genes in the proteomes of 10 *citrus* species. Initially, protein sequences were screened for the NB-ARC domain using the HMM model, resulting in the identification of 1875 sequences containing this domain (Table S1). Subsequently, 1845 sequences with the NB-ARC domain were confirmed via Blastp homology searches (Table S1). After redundancy reduction, sequences containing the NB-ARC or NAT domains were used to construct a phylogenetic tree, along with reported homologous sequences from representative plants and other species (including animals, bacteria, fungi, and archaea). We identified *citrus* proteins with the NB-ARC domain that formed monophyletic groups with plant R genes, designating these as *citrus* NLR protein sequences (Figure 1A). Some sequences with the NB-ARC domain formed monophyletic groups with non-plant homologs and were therefore excluded. Ultimately, we identified 1585 *NLR* genes (Table S1).

Utilizing the HMM method and the Pfam database, we annotated the domain structures of the 1585 *citrus NLR* genes, identifying 183 as TNL and 35 as RNL (Figure 1B). Notably, we also annotated the Rx_N (PF18052) domain, predicted to be a CC motif but suggested by research to be a four-helical bundle fold [34]. The Rx_N domain is equivalent to the CC domain annotated in previous studies [34]. Thus, in this context, RxN-NBS-LRR corresponds to CNL, as identified in prior research [12,35]. We collectively termed the NLR proteins containing the Rx_N domain as CNL, totaling 272 proteins (Figure 1B). Additionally, a substantial number of NL proteins containing only NBS or NBS-LRR domains were annotated, totaling 1095 (Figure 1B).

Among the 10 *citrus* species, aside from the 108 TNL sequences in C. *clementina*, the number of TNL sequences in the other 9 species was relatively low. Only a few were found in C. *medica*, C. *ichangensis*, C. *hindsii*, and C. *trifoliata*, while they were absent in C. *mangshanensis* and C. *grandis* (Figure 1B). RNL was the least abundant among the three types, evenly distributed across all species, ranging from three to four sequences each (Figure 1B). There were six RNL sequences in C. *sinensis* Osbeck cv. Newhall, possibly due to its diploid nature, resulting in a doubling of the quantity.

### 3.2. The Origin of Citrus NLR Genes

**Figure 2:**
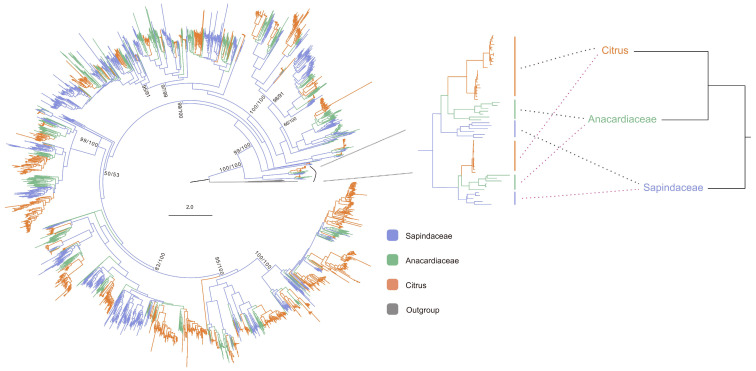
Phylogenetic analysis of NLR proteins in Sapindales. On the left, phylogenetic analysis was performed using an approximate maximum likelihood method with Q.mammal+R10. Proteins with MalT and SWACOS domains were used as outgroups. The SH-aLRT/bootstrap values are depicted near the selected nodes. The expanded section represents a part of the whole tree. The phylogenetic relationships between *Citrus*, Anacardiaceae, and Sapindaceae are based on Joyce [36]. Dashed lines indicate the one-to-one correspondence of *NLR* genes and species.

This study utilized 1585 identified NLR proteins and 1583 published NLR proteins from the Anacardiaceae and Sapindaceae families (Table S2) to construct phylogenetic relationships (Figure 2). The results indicate that *citrus* NLR proteins fall within the diversity of Sapindaceae, suggesting that *citrus NLR* genes originated comparatively later than those in Sapindaceae. Within the Sapindales order, Rutaceae is the oldest, followed by Sapindaceae, with Aurantioideae (*Citrus* genus) being the most recent [36]. In most monophyletic groups, the phylogenetic relationships of NLR proteins align with the species relationships of Rutaceae, Sapindaceae, and the *Citrus* genus (Figure 2). The coevolutionary relationships between NLR proteins and species suggest that *NLR* genes originated concurrently with the species’ origins.

### 3.3. The Evolution of Citrus NLR Genes

**Figure 3:**
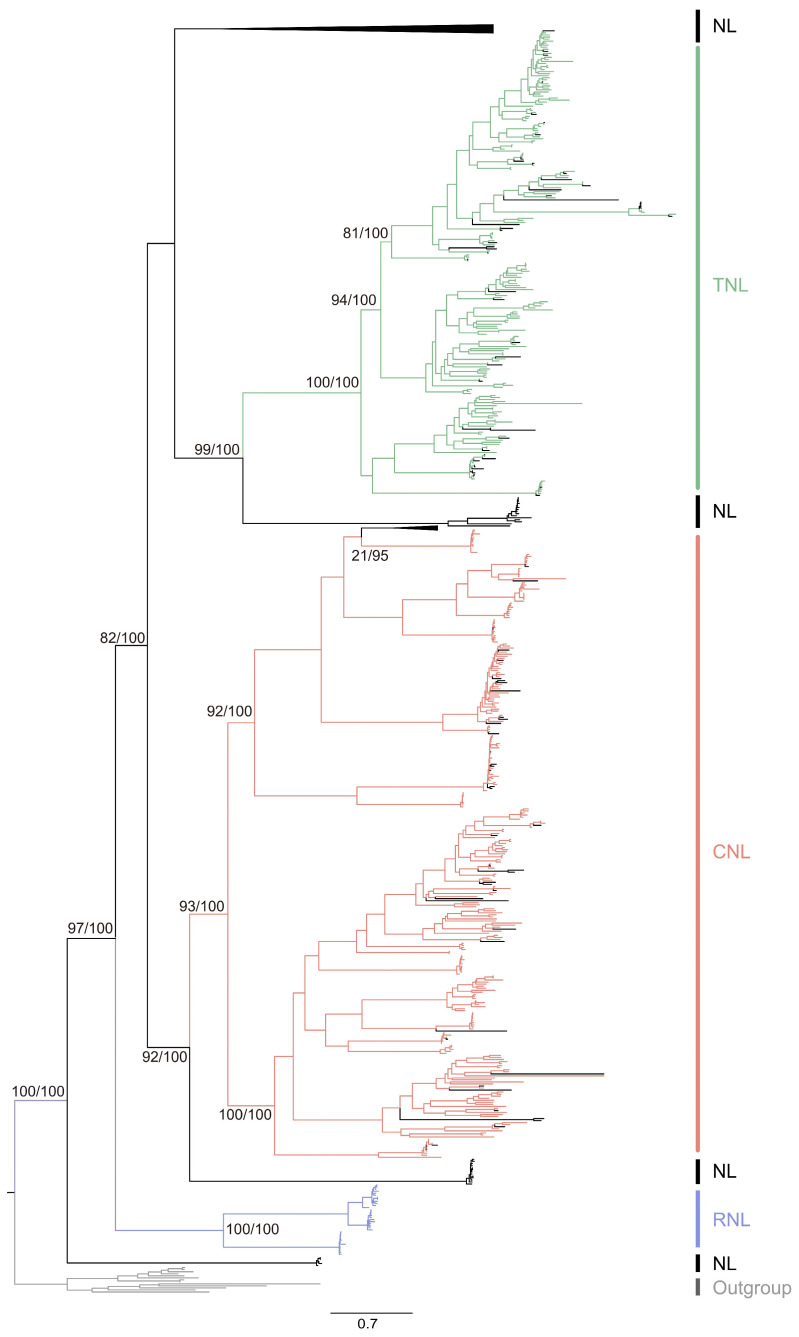
Phylogenetic analysis of *citrus* NLR proteins. The SH-aLRT/bootstrap values are depicted near the selected nodes. The green, red, purple, and black branches are TNL, CNL, RNL, and NL proteins, respectively. The NL clade is collapsed.

We employed maximum likelihood methods to construct phylogenetic relationships among *citrus* NLR proteins. Integrating the domain architecture of NLRs, we identified five main clusters in the phylogenetic tree of *citrus* NLR proteins, comprising one cluster of TNLs, one cluster of CNLs, one cluster of RNLs, and two clusters of NLs (Figure 3). NLs exhibited the highest diversity, while TNLs, RNLs, and CNLs fell within the diversity of NLs, indicating their origin from NLs with the acquisition of TIR, RPW8 domains, and CC motifs, respectively. Additionally, NLs were found within both TNLs and CNLs clusters (Figure 3), indicating TNLs and CNLs lost their TIR and Rx_N domains, respectively, transforming into NLs. Overall, *citrus NLR* genes underwent frequent events of domain acquisition and loss during evolution.

**Figure 4:**
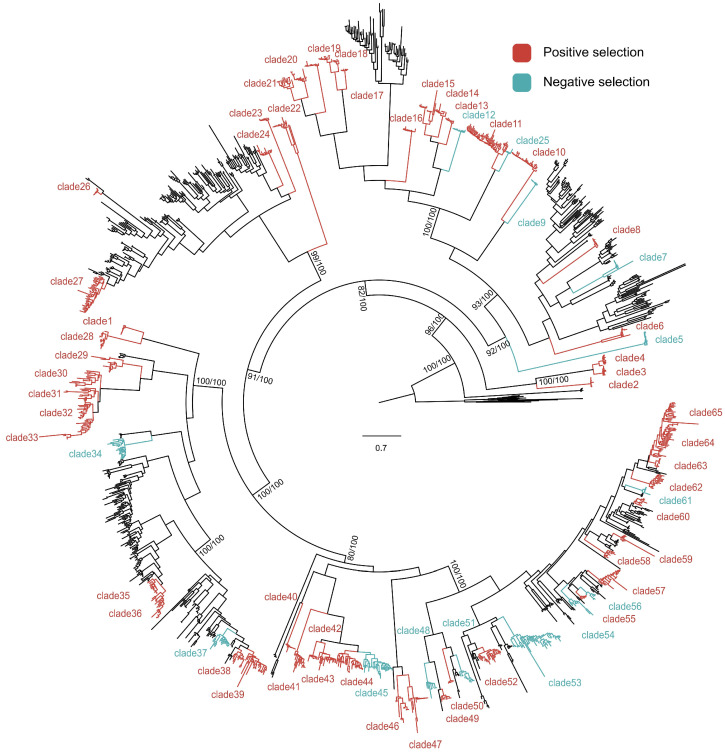
Positive selection analysis of *citrus NLR* genes. The phylogenetic tree is one shown in Figure 3. There are 65 groups for positive selection analysis as indicated. The red and green groups are detected NLR proteins containing amino acid residues under positive selection and negative selection, respectively.

To test whether *citrus NLR* genes underwent adaptive evolution after their origin, positive selection analysis was conducted on 65 groups selected based on phylogenetic tree. Amino acid residues under positive selection were detected in the majority of groups (52/65) (Figure 4). Among the 52 gene groups where positive selection was detected, the number of positively selected sites varied (Table S3). Our findings indicate that the vast majority of *citrus* NLR clusters underwent adaptive divergence.

### 3.4. Mechanism of NLR Gene Diversity Formation in Citrus

The phylogenetic tree of *citrus* was constructed using OrthoFinder2 software [29] based on the full genome of 10 *citrus* genomes (Figure 1A). *Atlantia buxifolia* is placed at the root of the tree, with the overall phylogenetic order largely consistent with previous reports [37]. Comparing the number of *NLR* genes across different *citrus* species on the phylogenetic tree reveals considerable diversity in NLR gene counts, even within the same subfamily. Among the 10 *citrus* species, substantial differences exist in the copy numbers of *NLR* genes. For instance, *C. clementina* has 405 copies, whereas *C. ichangensis* has only 56, making the former about 8 times more abundant than the latter. In the case of the sweet orange subspecies *C. sinensis* Osbeck cv. Newhall and *C. sinensis* Osbeck cv. Valencia, 178 and 107 copies were identified, respectively. Notably, while *C. sinensis* Osbeck cv. Newhall is a diploid genome, *C. sinensis* Osbeck cv. Valencia is a haploid genome. Although the genomic content of *C. sinensis* Osbeck cv. Newhall is double that of *C. sinensis* Osbeck cv. Valencia, the NLR gene copies are not proportionally higher. What evolutionary mechanisms have contributed to the genetic diversity of *citrus NLR* genes?

Based on the phylogenetic tree of *citrus* NLR proteins, we investigated horizontal gene transfer (HGT) and gene duplication events within *citrus NLR* genes. Gene duplication events were identified when *NLR* genes from the same species formed a monophyletic group on the phylogenetic tree. HGT events were identified when the genetic diversity of one species fell within that of another species.

**Figure 5:**
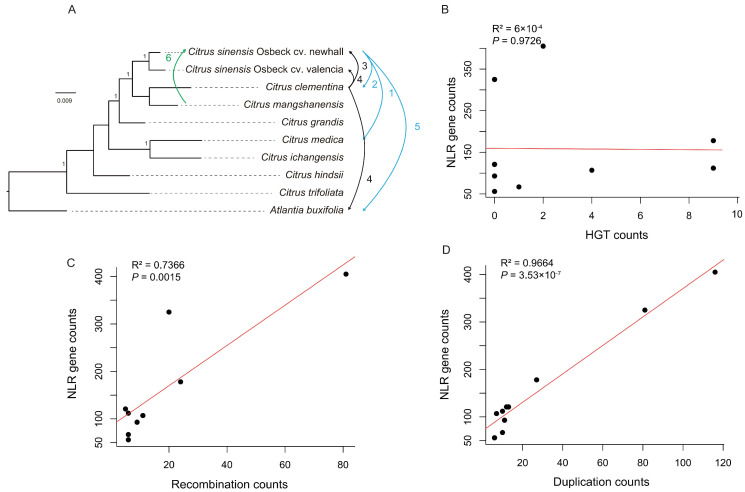
Mechanism analysis of NLR gene diversity. (**A**) Horizontal gene transfer events in *NLR* genes between *citrus* species. Arrows indicate the direction of transfer, and numbers indicate the number of HGT events. (**B**) The relationship between the number of *NLR* genes and HGT events. (**C**) The relationship between the number of *NLR* genes and gene recombination events. (**D**) The relationship between the number of NLR genes and gene duplication events.

Following statistical analysis, we generated a diagram depicting the levels of HGT between various *citrus* species (Figure 5A), revealing relatively frequent gene exchange among *C. sinensis* Osbeck cv. Newhall, *C. sinensis* Osbeck cv. Valencia, *C. mangshanensis*, and *C. clementina*. Notably, we observed gene exchange between *C. sinensis* Osbeck cv. Newhall and *C. clementina* with *Atlantia buxifolia*. Additionally, there was minimal gene exchange between *C. sinensis* Osbeck cv. Newhall and *C. medica*, while the remaining four *citrus* species (*C. ichangensis*, *C. hindsii*, *C. trifoliata*, and *C. grandis*) and other species showed no *NLR* gene exchange. Correlation analysis between the number of *NLR* genes and the frequency of HGT events revealed a non-significant positive relationship (Figure 5B), suggesting that horizontal gene transfer contributes insignificantly to *citrus* NLR gene diversity.

The number of gene duplications and recombinations in *C. medica* and *A. buxifolia* was the same, with 10 and 6 instances, respectively. HGT occurred nine times in *A. buxifolia* and only once in *C. medica*. The NLR gene copy number in *A. buxifolia* was 1.67 times that in C. *medica*. Therefore, HGT is the primary mechanism driving the increase in NLR gene copy number in *A. buxifolia*.

Recombination has long been considered a significant driver for NLR gene diversification [38]. We used seven algorithms within the RDP 5.05 software [32] to search for recombination signals within *citrus NLR* genes. A single gene recombination event was considered when four or more algorithms detected positive signals. In this study, 173 recombination events were identified, with C. *clementina* exhibiting the highest number at 81 occurrences (Table S4). Correlation analysis between the number of *NLR* genes and the number of recombination events across *citrus* species showed a significant positive relationship (Figure 5C), indicating that gene recombination is one of the mechanisms contributing to *citrus* NLR gene diversity.

A total of 293 gene duplication events were recorded in this study, with occurrences ranging from 6 to 116 across different *citrus* species. *C. clementina* once again exhibited the highest number of gene duplication events at 116 occurrences (Table S4). Correlation analysis between the number of *NLR* genes and the number of gene duplication events revealed a positive relationship, indicating that gene duplication also contributes to *citrus* NLR gene diversity.

The statistical analysis of recombination, HGT, and gene duplication reveals that *Citrus clementina* experienced gene duplication and recombination events as high as 116 and 81, respectively, resulting in a substantial increase in copy number. In contrast, *Citrus ichangensis* underwent only six instances of gene duplication and recombination, leading to a limited copy number. Thus, the stark difference in copy number between *Citrus ichangensis* and *Citrus clementina* can be attributed to the disparity in gene duplication and recombination events. In summary, gene recombination and gene duplication are the main mechanisms shaping NLR gene diversity in *citrus*.

## 4. Discussion

In this study, we employed comparative genomics and phylogenetic methods to identify *NLR* genes across 10 *citrus* species. To validate the effectiveness of our mining approach, we used NLR-Annotator software [28] to search nucleotide genomes, resulting in the retrieval of 1847 *citrus* NLR gene sequences. The NLR counts extracted by NLR-Annotator across various *citrus* species closely matched the results obtained from Blastp and HMM of this study. Comparison with previously reported *citrus* NLR gene numbers in the ANNA database [12] revealed close correspondence between the quantities in *A. buxifolia*, *C. grandis*, *C. medica*, and *C. ichangensis* and the results obtained in this study through Blastp and HMM. Thus, the identification methods utilizing Blastp and HMM employed in this study are validated.

The ANNA database reports 529 *NLR* genes of C. *sinensis* Osbeck cv. Valencia [12], whereas our study’s Blastp and HMM analyses yielded 179 and 178, respectively, and the NLR-Annotator software result was 181. The substantial variance can be attributed to the utilization of the C. *sinensis* Osbeck cv. Valencia v2.0 genome [39] in prior studies, while we employed the v3.0 genome version [40]. Factors such as genome assembly quality and annotation precision between the two genome versions may influence the NLR gene number. We conducted NLR gene mining on the nucleotide sequences of both sweet orange genome versions using the tblastn algorithm. We found comparable outcomes, with no significant differences between the two genome versions. This underscores annotation precision as the primary factor influencing the quantity of *NLR* genes.

We integrated the results from Blastp and HMM, eliminating redundancies. Subsequently, we employed phylogenetic analysis to further screen the sequences, remove false positives, and discard NLR sequences generated by alternative splicing, thereby obtaining the NLRs for each *citrus* species in this study. We employed a more stringent identification method to ensure the accuracy of the results.

The distribution of TNLs varies significantly among *citrus* species. TNLs are sparsely present or absent in *C. medica*, *C. ichangensis*, *C. hindsii*, *C. trifoliata*, *C. grandis*, and *C. mangshanensis*, while in *C. clementina*, their number can be as high as 108. To exclude the impact of gene annotation on the results, we used the tblastn algorithm to mine the genomes of *C. medica*, *C. ichangensis*, *C. hindsii*, *C. trifoliata*, *C. grandis*, and *C. mangshanensis* for TNLs. The identification results were consistent with our study, thus eliminating the influence of genome annotation on the identification results. The evolutionary fate of *NLR* genes is mainly determined by natural selection and genetic drift [21]. In this study, positive selection amino acid residues were detected in all four clusters of TNLs selected, suggesting that the loss of TNLs in species such as *C. mangshanensis* may be due to genetic drift.

In-depth analysis revealed that the expansion of TNL gene copies in *C. clementina* is due to frequent gene recombination and duplication events. On one hand, previous studies have speculated multiple origins of TNLs [41], while loss has been reported in Aquilegia coerulea and monocots [42]. On the other hand, research has indicated that the evolutionary rate of TNLs is higher than that of non-TNLs [41], suggesting that, compared with CNLs and RNLs, TNLs may be more prone to acquiring or losing the TIR domain, thus undergoing a more complex evolutionary process.

Differences in NLR gene copy numbers among *citrus* species are not uncommon, as seen in the Rosaceae family, where the wild strawberry (*Fragaria vesca*) has 144 copies, while the apple (*Malus pumila*) has 748 copies [41]. This study further analyzes gene recombination and duplication as the primary mechanisms shaping NLR gene diversity in *citrus*. While HGT did not exhibit significant impact across all *citrus* species, it was the major driver of the NLR gene copy number increase in *A. buxifolia*. The number of gene duplications and recombinations in *C. medica* and *A. buxifolia* was the same, with 10 and 6 instances, respectively. HGT occurred nine times in *A. buxifolia* and only once in *C. medica*. The NLR gene copy number in *A. buxifolia* was 1.67 times that in C. *medica*. Therefore, HGT is the primary mechanism driving the increase in NLR gene copy number in *A. buxifolia*.

Previous studies have indicated that the reduction in *NLR* genes in angiosperms is linked to ecological specialization and the loss of signal transduction components [12]. This contraction of *NLR* genes has been associated with adaptations to aquatic, parasitic, and carnivorous lifestyles [12]. The convergent reduction of NLRs in aquatic plants bears resemblance to the absence of NLR expansion throughout the prolonged evolutionary history of green algae prior to their terrestrial colonization [12]. Among *citrus* species, *Citrus sinensis* and *Citrus clementina* are the most widely cultivated, thriving in complex environments, which may account for their relatively higher copy number of RNL genes.

Approximately 30 million years ago, the orange subfamily (Rutaceae: Aurantioideae) originated from the ancient Indian plate, which was then connected to Africa and Australia. As the plates collided and drifted, the Rutaceae subfamily gradually diversified, with the *citrus* genus originating over 8~9 million years ago in southern China [36]. During the colonization of terrestrial environments, plants encountered new pathogen species and infection levels [43]. Positively selected *NLR* genes play a crucial role in plant–pathogen interactions. This evolutionary process involved frequent gains and losses of TIR, RPW8, and LRR domains, enhancing R-protein diversity and enabling adaptation to various environmental challenges [13]. The discovery of numerous adaptively evolved *NLR* genes in *citrus* suggests their significant role in global *citrus* colonization.

## 5. Conclusions

This study provides a comprehensive understanding of the diversity and evolution of *NLR* genes in *citrus* species, which were previously underexplored compared with other plants. This study identified 1875 *NLR* genes across 10 *citrus* species and analyzed their variations, origins, and adaptive evolution. The findings are particularly significant for their potential application in breeding disease-resistant *citrus* varieties, making this study a foundational resource for future *citrus* genetic research and crop improvement efforts.

## Data Availability

The amino acid sequence of NLR protein in *citrus* species is accessible in the Science Data Bank (https://doi.org/10.57760/sciencedb.11629).

## Supplementary Materials

The following supporting information can be downloaded at: https://www.mdpi.com/article/10.3390/biology13100822/s1, Table S1: The identification of *citrus NLR* genes; Table S2: This study used *NLR* genes of Anacardiaceae and Sapindaceae; Table S3: Positive selection analysis of NLR gene groups in *citrus*; Table S4: Statistics of three events occurring in *citrus NLR* genes. Figure S1: Phylogenetic analysis of *citrus* NLR proteins. The Clades boxed by the green box is selected for the positive selection analysis.
