# Supplementary material for: Diversity and Evolution of NLR Genes in Citrus Species
Source: Biology (Basel). 2024 Oct 14;13(10):822. doi: 10.3390/biology13100822 (PMC11504038; doi:10.3390/biology13100822)
Supplement: Supplementary file 1 [file biology-13-00822-s001.zip › biology-3222647-Tables.pdf]

Table S1: the identification of citrus NLR genes

**Species**	**NBS domain-containing protein (Blastp)**	**NBS domain-containing protein (HMM)**	**NLR**	**NLR Annotator**	**ANNA database**
C. clementina	412	420	405	434	429①
C. mangshanensis	366	389	325	320	-
C. sinensis Osbeck cv. newhall	183	193	178	204	-
C. sinensis Osbeck cv. valencia	179	178	107	181	529①
C. hindsii	122	124	93	129	-
Atlantia buxifolia	146	121	112	125	131①
C. grandis	124	129	121	119	132①
C. trifoliata	174	170	121	170	-
C. ichangensis	64	70	56	72	74①
C. medica	75	81	67	93	84①
Total	1845	1875	1585	1847	-

Note: ①: the data got from ANNA database (Liu et al., 2021)

Table S2: The study used NLR genes of Anacardiaceae and Sapindaceae

**Family**	**Species**	**NO. of NLR genes**
Anacardiaceae	*Mangifera indica*	107①
	Pistacia vera	367①
	Sclerocarya birrea	146①
Sapindaceae	*Acer yangbiense*	248①
	Dimocarpus longan	541①
	Xanthoceras sorbifolium	174①

Note: ①: the data got from (Liu et al., 2021)

Table S3: Positive selection analysis of NLR gene groups in citrus

Group number	Type	Sequence counts	Pvalue M7:M8	Positively selected sites with P > 0.95
1	NL	10	6.99E-06	4D 45T 161P 626I
2	RNL	12	0.001381143	840H 871L 872H
3	RNL	11	0.000156005	78E 363T 505I 506K 508R 515T
4	RNL	12	2.43E-08	5F 44R 49W 78E 649R
5	NL	13	0.9853425	-
6	NL	10	3.67E-06	1013H 1094F
7	CNL	10	0.2018214	-
8	CNL	10	2.33E-11	194K 199F 873R 966A 1205K
9	CNL	8	0.2866223	-
10	CNL	28	1.28E-13	350G 578R 654R 683E
11	CNL	40	0	34L 205D 279N 738G 740S 746R 915R 943S 974K 1071S 1072G 1073S 1074S 1075I 1077E 1106V 1131L 1145T 1147E 1149T 1173T 1189L 1195D 1205R 1217R 1219A 1221T 1239I 1241N 1243L 1244D 1246V 1266R 1289L 1291V 1293S 1294C 1313E 1315I 1316P 1317D 1321I 1323V 1324K 1327E 1328V 1330H 1332R 1335V
12	CNL	12	0.2091148	-
13	CNL	8	1.37E-05	88A 106S 113K 471E 540I 849M
14	CNL	10	5.51E-13	249A 312S 314N 315I 317S 325R 332C 334Y 338Q 584Q 624E 628Q 630E 658I 661E 686N 747S 757D 830S 844D 845I 846H 847V 848I 882S 892F 955T
15	CNL	14	0.000463903	944K
16	CNL	12	0.000112996	21F 564R 565S 744V 919V
17	NL	6	0.8676239	-
18	NL	15	0	25E 36E 56K 125L 130G 134T 136D 140R 536H 579K 582E 759D 907H 913R 937E 941Y 943E 1087T 1157A
19	NL	11	1.48E-08	838P 911M 924K 939W 942K 1012V 1018I
20	NL	11	0	64N 859E
21	NL	12	0	2G 4Q 5A 6F 24G 37S 42S 46F 57V 62L 66Q 121A 159H 160H 161V 162D 163F 164W 171R 409V 410A 446G 479L 720P 725P 727E 728F 741P 753E 770E 773G 775E 776Y 777G 794H 795S 796L 798D 801S 820L 821V 822H 823D 825M 827P 828L 829E 842G 847G 852K 872E 874T 934V 938K 942S 945L
22	NL	15	1.01E-06	1092C 1440C

23	TNL	8	0	277E 628H 1209L 1267S 1272C 1275S 1279G 1281Q
24	TNL	13	3.34E-09	878I 985N
25	CNL	5	0.0104684	-
26	TNL	6	0	392S 400E 499S 511E 512S 522S 534A 535K 539S 1698T 1849G 1851R 1854I
27	TNL	33	0	115G 158S 161N 170F 174S 178Q 196Q 775S 802D 804H
28	NL	17	2.04E-12	25R 300K 1050L 1051E 1064L 1078W 1079E
29	NL	15	2.28E-09	931K 971R 1007N 1018V 1022C
30	NL	15	0	22P 285E 459A 460K 507S 509V 510L 520V 523G 525A 572K 637S 680G 742N 752E 754D 776D 792P 802Y 804H 834R 839L 867K 932L 938I 956C 958R 959D 982R 985V 1011K 1014R 1041E 1043E 1070F 1072T 1098R 1100R 1128E 1130S 1133Y 1153T 1156Q 1159Q 1224E
31	NL	12	0	80K 336S 414S 637K 657G 812D 837N 838Y
32	NL	15	0	451G 563A 565W 568E 570G 571L 574Y 593C
33	NL	16	0	398K 628I 666S 669L 671C 677D 679K 703V 709S 714R 717L 718R 719G 727S 728S 732D 736D 737R 738F 743D 746R 797S 798E 808V 812A 819V 824K 830S 833G 844E 857E 874A 908T 909D 911S 912F 913L 915K 917E 961V 992P 1380S 1383K
34	NL	20	0	771A 772E 797F 798S 804Y 806L 808S 813L 818H 824F 826R 853E 939Q 940D 951I 954R 962R 963W 969E 970D 972R 974T 1002R
35	NL	13	0	67I 112V 113K 120I 175S 309H 310K 311G 312C 316L 318A 321Q 323V 325S 326G 328M 332P 333N 343E 346S 350K 352A 353G 355Y 357E 358G 359S 361F 362Q 363S 364I 367D 369A 372C 377V 379I 381T 382I 391L 397A 400Q 406S 407T 415P 417A 420A 426N 427K 429E 430G 431E 433L 441G 444A 446S 447S 448I 460L 462Q 467M 469V 472A 473R 474V 475L 476T 494K 499S 505R 506D 507V 508A 509I 510S 513S 514R 516Q 517N 522T 531E 558V 559E 566L 569S 575S 576S 577L 578T 591R 595L 596T 600L 601L 602S 607L 611L 615A 619C 621C 625L 635G 636G 638K 639K 644C 653L 654P 655T 660L 662W 666L 668L 669R 672R 676V 684N 687S 690S 691H 693E 696Y 698S 703Q 718E 719E 732D 734I 737V 741L 749L 750E 751K 755Y 756R 782I 790V 793Q 794G 795I 798L 800L 803L 804Q 810Y 816A 819G 822E 825F 831G 834D 841E 842S 846R 878Y
36	NL	16	1	-
37	NL	15	0	226D 561D 630L 1072P 1188S 1193G

38	NL	11	0	6V 899W 920R 924R 925S 928D 1021G 1022M 1023H 1 024T 1025W 1026E 1049W 1050P 1051A 1053E 1054T 1 055L 1056V 1058Y 1059G 1061D 1070D 1071L 1072S 1 073Q 1075N 1076G 1077N 1078D 1103G 1105P 1107Q 1 108Q 1110L 1111L 1114E 1115K 1116V
39	NL	21	0.8077772	-
40	NL	11	1.81E-12	15G 45E 46V 83I 101Y 257L 384C 410C 414C 418P 42 1F 422S 425E 427E 428L 431Y 436G 438I 440E 441Q 4 43N 444H 445 449N 452I 456E 457N 465E 466D 469N 472T 473V 474K 476H 477D 482V 485W 486I 488S 490 L 492N 494C 500S 998K 1007G 1015D
41	NL	14	0	160G 211R 212S 221L 224E 227T 280E 311Y 345S 347 D 370A 434L 435A 450A 483R 507E 516N 531S 551E 553N 554Y 578L 580R 622Y 624G 646A 647I 729K 731 L 757M 759C 760W 762K 784S 786L 787G 827Y 828R 851T 853N 854N 894F 896N 897L 918T 920T 922L 923 R 949E 951D 953G 977R 979W
42	NL	9	6.56E-08	160D 187Q 189E 190T 202S 219N 220Q 221N 227N 834 M
43	NL	18	0	270G 284P 329E 375V 509P 576R 602A 681T 706A 709 T 712Y 717T 718R 720A 727Y 732C 737V 738T 740V 774K 923D
44	NL	26	0	89P 131S 179P 203R 289S 334L 380S 509R 536G 537L 540Q 559K 561N 574D 583A 585G 586N 608H 630R 63 1Y 633L 634R 641V 654A 656R 676Y 678L 679P 700S 703R 722T 729T 1329S 1330D 1333G 1346S 1348M 134 9N 1358P 1367F 1369Q 1371F 1372E 1374S 1403S 1405 V 1406R 1408P 1429K 1431K 1432V 1434F 1469G 1471 Y 1472F 1474P 1497R 1498G 1527E 1541A 1554V 1555 G 1556F
45	NL	28	0	235N 402F 417S 475H 487A 603P 610H 668E 678L 682 E 700E 702H 724R 726V 727H 749H 771R 772W 774G 794E 795A 797R 817Y 819W 820R 844R 863Y 867C 87 0K 890G 892A 895A 896H 897G 898M 899G 900G 901 Y 902L 999P 1009F 1010L 1011Y 1012G 1021T 1032R 1034Q 1036F 1037A 1039D 1068R 1070A 1071N 1073R 1094K 1096Y 1097N 1134G 1136Y 1137Y 1160E 1162R 1163R 1186H 1192R 1217A 1231E 1232R
46	NL	11	5.61E-07	74R 302K 309E 818V 853I
47	NL	17	0.4310953	-
48	NL	8	1.37E-13	14C 917Q 919I
49	NL	9	0.00210002	906S

			3	
50	NL	6	0.45405	-
51	NL	11	4.00E-15	194F 198I 224A 529R 539V 590E 593A 596K 610L 613Q 614T 617Q 618A 641Y 642K 643Y 677F 678C 699S 703S 751D 777V
52	NL	21	0	66N 70P 85K 179N 245D 431E 521D 542E 543Y 567G 569D 591W 616Y 619R 639C 643S 701H 723E 725D 727E 750S 752A 754R 755N 778H 808A 812L 814I 836T 838S 840S 841L 863Y 888L 890L 891R 892I 893M
53	NL	40	0	86T 118K 176K 203C 208H 235W 305V 373K 376R 425E 437D 510I 514G 516R 518D 536A 538S 552S 560S 563A 584W 586E 608A 609Y 610M 631L 632G 638S 639N 642T 676R 698V 700Y 701Q 703E 704C 727H 729E 730D 731S 732D 759S 761E 762N 764E 765K 766M 780E 782E 784S 787H 788A 794S 824A 826N 828F 829N 831V 850Q 852Y 853S 875E 877T 879K 899V 945V 946L 947P 948E 949Y 950L 951D 952Y
54	NL	8	1.92E-09	44R 442D 475G 533Q 540R 616S 665L 675F 676K 724S 92L 948S 949
55	NL	6	0.3052189	
56	NL	6	0.9889653	-
57	NL	20	0	7V 100A 110I 560M 581L 655S 741D 795A 830H 832S 883Q 885E
58	NL	9	0.9207847	-
59	NL	7	0	369Y 507Q 533L 534I 535T 536L 537F 538L 539G 541N 542M 543L 571L 573S 574G 575I 578L 579V 580S 581L 582Q 583Y 584L 585N 586L 588E 589T 590F 591I 593E 594L 596H 597E 598L 600A 601L 602K 604L 606C 607L 609L 611Y 612M 615L 616H 617T 619P 620R 621Q 622L 623L 624C 626F 627S 628G 630K 632L 637C 638G 642T 644P 648V 649L 651G 653S 656L 657V 658E 660L 662T 663L 664E 669L 670S 672T 684S 685C 686Q 687Q 688Y 690S 691C 692K 694A 696E 699R 701E 703S 713D 714L 715K 718D 721D 727S 729E 730E 733V 735Y 736A 738V 739Q 741T 742R 743E 744L 745M 746V 747S 748T 749A 750F 752V 754P
60	NL	8	3.79E-13	551Q 650G 707C 713S 731A 761V
61	NL	6	0.05597148	712K
62	NL	14	0	19P 40T 74V 123A 214S 347Q 357G 463G 524V 531R 535D 553R 555P 579E 580T 601N 605T 626K 648F 652F 660L 661L 664W 665H 708L 710H 711R 713D 733D 735F 736S 740I 741K 769R 770F 792S 793Y 827E 829G

				832V 833R 851Q 853W 854V 878G 880Y
63	NL	14	0.0116052	-
64	NL	50	1	256R 260A 281G 284Q 521S 730G 765E 766D 768E 788N 790G 791K 792S 794S 795N 800V 816H 818S 820R 828K 841E 842L 1806L 1808H 1810A 1811R 1814T 1832W 1834D 1835R 1837K 1868H 1869I 1871Q 1876T 1889S 1891F 1892H 1894G 1923L 1925R 1927S 1928H 1931N 1936Y 1950V 1953R 1954G 1959E 1979E 1980E 1981D 2003P 2004R 2005S
65	NL	16	0	353V 555I 563R 564E 566K 592K 616H 618C 620Q 628L 641I 642W 667W 668F 669S 726F 728Q 730F 752C 754S 755N 788E 789E 791H 796L 809S 811Y 812D 814G 847M 848A 851E 869E 871L 872G 896D 897T 898Q 920T

Table S4: Statistics of three events occurring in citrus NLR genes

Species	Recombination	HGT	Gene duplication
Citrus sinensis Osbeck cv. newhall	24	9	27
Citrus sinensis Osbeck cv. valencia	11	4	7
Citrus clementina	81	2	116
Citrus mangshanensis	20	0	81
Citrus grandis	5	0	13
Citrus medica	6	1	10
Citrus ichangensis	6	0	6
Citrus hindsii	9	0	11
Citrus trifoliata	5	0	12
Atlantia buxifolia	6	9	10
Total	173	25	293
