# Supplementary figures and images for: Diversity and Evolution of NLR Genes in Citrus Species
Source: Biology (Basel). 2024 Oct 14;13(10):822. doi: 10.3390/biology13100822 (PMC11504038; doi:10.3390/biology13100822)

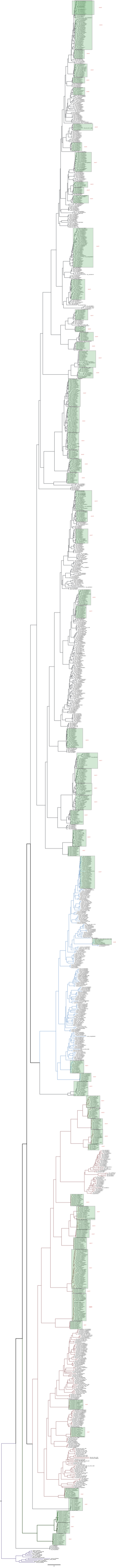

Supplement: Supplementary file 1 [file biology-13-00822-s001.zip › biology-3222647-Figure S1.pdf]
